# Crystal structures of three anhydrous salts of the Lewis base 1,8-di­aza­bicyclo­[5.4.0]undec-7-ene (DBU) with the ring-substituted benzoic acid analogues 4-amino­benzoic acid, 3,5-di­nitro­benzoic acid and 3,5-di­nitro­salicylic acid

**DOI:** 10.1107/S205698901600267X

**Published:** 2016-02-20

**Authors:** Graham Smith, Daniel E. Lynch

**Affiliations:** a Science and Engineering Faculty, Queensland University of Technology, GPO Box 2434, Brisbane, Queensland 4001, Australia; b Exilica Ltd, The Technocentre, Puma Way, Coventry CV1 2TT, England

**Keywords:** crystal structure, 1,8-di­aza­bicyclo­[5.4.0]undec-7-ene, BDU, benzoate salts, hydrogen bonding

## Abstract

The anhydrous salts of the Lewis base 1,8-di­aza­bicyclo­[5.4.0]undec-7-ene (DBU) with 4-amino­benzoic acid [1-aza-8-azoniabi­cyclo­[5.4.0]undec-7-ene 4-amino­benzoate, C_9_H_17_N_2_^+^·C_7_H_6_NO_2_^−^ (I)], 3,5-di­nitro­benzoic acid [1-aza-8-azoniabi­cyclo­[5.4.0]undec-7-ene 3,5-di­nitro­benzoate, C_9_H_17_N_2_^+^·C_7_H_3_N_2_O_6_^−^, (II)] and 3,5-di­nitro­salicylic acid (DNSA) [1-aza-8-azoniabi­cyclo­[5.4.0]undec-7-ene 2-hy­droxy-3,5-di­nitro­benzoate, C_9_H_17_N_2_^+^·C_7_H_3_N_2_O_7_^−^, (III)] have been determined and their hydrogen-bonded structures are described. In both (II) and (III), the DBU cations have a common disorder in three of the C atoms of the six-membered ring moieties [site-occupancy factors (SOF) = 0.735 (3)/0.265 (3) and 0.686 (4)/0.314 (4), respectively], while in (III), there is additional rotational disorder in the DNSA anion, giving two sites (SOF = 0.72/0.28, values fixed) for the phenol group. In the crystals of (I) and (III), the cation–anion pairs are linked through a primary N—H⋯O_carbox­yl_ hydrogen bond [2.665 (2) and 2.869 (3) Å, respectively]. In (II), the ion pairs are linked through an asymmetric three-centre *R*_1_^2^(4), N—H⋯O,O′ chelate association. In (I), structure extension is through amine N—H⋯O_carbox­yl_ hydrogen bonds between the PABA anions, giving a three-dimensional structure. The crystal structures of (II) and (III) are very similar, the cation–anion pairs being associated only through weak C—H⋯O hydrogen bonds, giving in both overall two-dimensional layered structures lying parallel to (001). No π–π ring associations are present in any of the structures.

## Abstract 2

The anhydrous morpholinium salts of 1,8-di­aza­bicyclo­[5.4.0]undec-7-ene (DBU) with 4-amino­benzoic acid, 3,5-di­nitro­benzoic acid and 3,5-di­nitro­salicylic acid, provide one example of a three-dimensional hydrogen-bonded network polymer and two of weakly inter-associated hydrogen-bonded cation–anion units.

## Chemical context and database survey   

The Lewis base 1,8-di­aza­bicyclo­[5.4.0]undec-7-ene (DBU) is an alkaloid isolated from the sponge *Niphates digitalis* (Regalado *et al.*, 2010 ▸) but is commonly synthesized. It finds use as a curing agent for ep­oxy resins, as a catalyst in organic syntheses, and as a counter-cation in metal complex chemistry, e.g. with the penta­bromo­(tri­phenyl­phosphane)platinum(IV) monoanion (Motevalli *et al.*, 1989 ▸). It has also found use in binding organic liquids (BOLs), which usually comprise a mixture of amidines or guanidine and alcohol, and are used to reversibly capture and release gases such as CO_2_, CS_2_, SO_2_ or COS (Shannon *et al.*, 2015 ▸; Pérez *et al.*, 2004 ▸; Heldebrant *et al.*, 2009 ▸). The structure of one of these formed from the absorption of CO_2_ is the bicarbonate (Pérez *et al.*, 2004 ▸).

As a very strong base (p*K*_a_ *ca* 14), protonation of the N8 group of the six-membered hetero-ring of DBU is readily achieved and results in the formation of salts with carb­oxy­lic acids and phenols. The Cambridge Structural Database (2015 version) (Groom & Allen, 2014 ▸) contains 35 examples of organic salts of DBU, among them the benzyl di­thio­carbonate (Heldebrant *et al.*, 2009 ▸) and the phenolate from 2,6-di(*tert*-but­yl)-4-nitro­phenol (Lynch & McClenaghan, 2003 ▸). However, of the total there are surprisingly few carboxyl­ate salts, *e.g.* with Kemp’s triacid (1,3,5-tri­methyl­cyclo­hexane-1,3,5-tri­carb­oxy­lic acid) (a monoanionic aceto­nitrile salt) (Huczyński *et al.*, 2008 ▸) and the dianionic salt of the tetra­(3-carb­oxy­phen­yl)-substituted porphyrin (Lipstman & Goldberg, 2013 ▸).

No reported crystal structures of salts with simple substituted benzoic acids are found, so in order to examine the hydrogen-bonding in crystals of the DBU salts with some common ring-substituted benzoic acids, a number of these were prepared. Suitable crystals were obtained with 4-amino­benzoic acid (PABA), (3,5-di­nitro­benzoic acid (DNBA) and (3,5-di­nitro­salicylic acid (DNSA), giving the anhydrous salts, C_9_H_17_N_2_^+^ C_7_H_6_NO_2_^−^ (I), C_9_H_17_N_2_^+^ C_7_H_3_N_2_O_6_^−^ (II) and C_9_H_17_N_2_^+^ C_7_H_3_N_2_O_7_^−^ (III), respectively and their structures and hydrogen-bonding modes are reported herein.
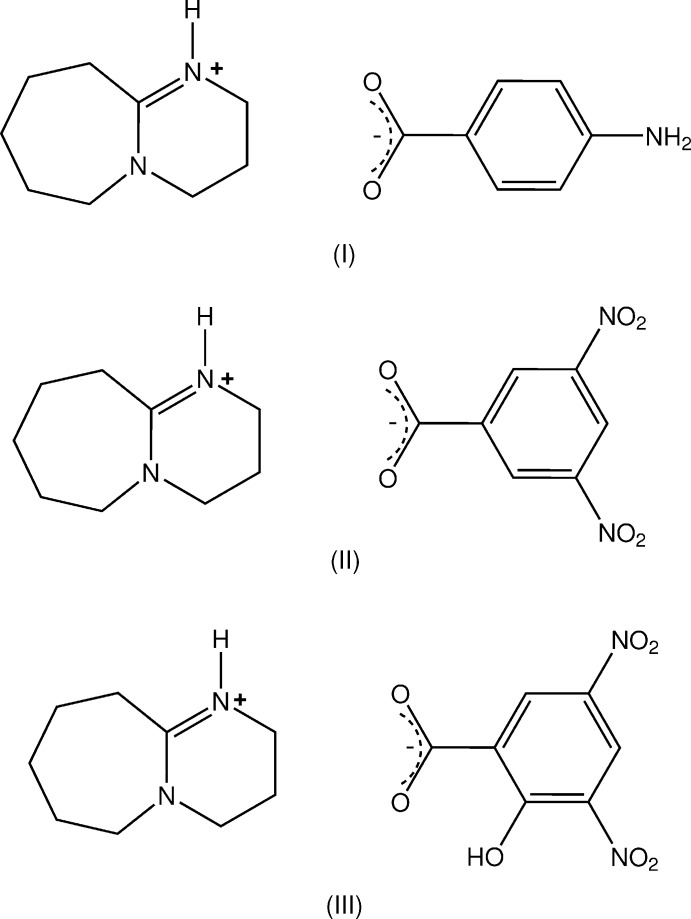


## Structural commentary   

**Figure 1:**
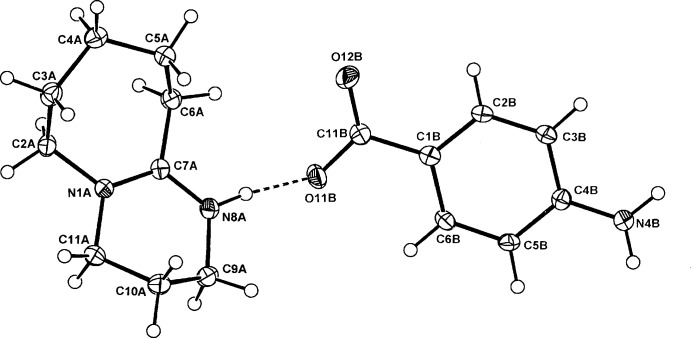
The atom-numbering scheme and the mol­ecular conformation of the DBU cation (*A*) and the PABA anion (*B*) in (I) with displacement ellipsoids drawn at the 40% probability level. The cation–anion hydrogen bond is shown as a dashed line.

**Figure 2:**
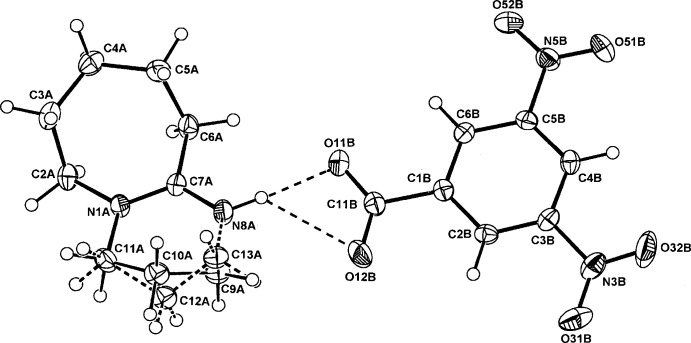
The atom-numbering scheme and the mol­ecular conformation of the DBU cation (*A*) and the DNBA anion (*B*) in (II) with displacement ellipsoids drawn at the 40% probability level. The bonds in the minor disordered section of the six-membered ring of the cation and the cation–anion hydrogen bonds are shown as dashed lines.

**Figure 3:**
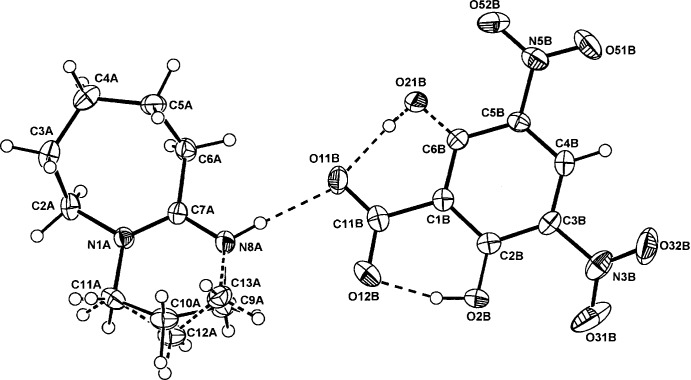
The atom-numbering scheme and the mol­ecular conformation of the DBU cation (*A*) and the DNSA anion (*B*) in (III) with displacement ellipsoids drawn at the 40% probability level. The bonds in the minor disordered section of the six-membered ring of the cation are shown as dashed lines.

The asymmetric units of (I)–(III) comprise a BDU cation (*A*) and a 4-amino­benzoate anion (*B*), (I) (Fig. 1 ▸), a 3,5-di­nitro­benzoate anion (*B*), (II) (Fig. 2 ▸), and a 3,5-di­nitro­salicylate anion (*B*), (III) (Fig. 3 ▸). The cation–anion pairs in (I) and (III) are linked through a primary N8*A*—H⋯O_carbox­yl_ hydrogen bond [2.665 (2) and 2.871 (3) Å, respectively; Tables 1 ▸ and 3 ▸]. In (II), the ion pairs are linked through an asymmetric three-centre 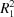
(4), N8*A*—H⋯*O,O′* chelate association [2.777 (2), 3.117 (2) Å; Table 2 ▸]. With (III), the corresponding longer contact with the second carboxyl O12*B* atom is 3.222 (3) Å (Fig. 3 ▸).

With the structures of (II) and (III), there is disorder in the six-membered ring system involving atoms C9*A* and C10*A* (with alternative minor occupancy sites C12*A* and C13*A*), giving similar site occupancy factors [SOF 0.735 (3)/0.265 (3) and 0.686 (4)/0.314 (4) for (II) and (III), respectively]. This feature is found in three other structures among the CSD set: the previously mentioned 2,6-di(*tert*-but­yl)-4-nitro­phenolate (SOF 0.60/0.40) (Lynch & McClenaghan, 2003 ▸); in the 8-bromo­guanosine 8-bromo­guanoside adduct salt (SOF = 0.63/0.37) (Saftić *et al.*, 2012 ▸) and in the counter-cation of a bromo­carbyne Mo complex (SOF = 0.83/0.17) (Cordiner *et al.*, 2008 ▸).

With the PABA anion in (I), the carboxyl­ate group is essentially coplanar with the benzene ring [torsion angle C2*B*—C1*B*—C11*B*— O11*B* = 179.25 (15)°, a feature similar to those found in the parent acid (Gracin & Fischer, 2005 ▸) and its co-crystals, *e.g.* with 4-nitro­benzoic acid (Bowers *et al.*, 2005 ▸).

The carboxyl­ate groups of the DNBA and DNSA anions in both (II) and (III) are also essentially coplanar with the benzene rings: torsion angles C2*B*—C1*B*—C11*B*—O11*B* = −176.60 (16) and −179.4 (2)°, respectively. The 5- and 3-substituted nitro groups are also either in-plane or out-of-plane [torsion angles C4*B*—C5*B*—N5*B*— O52*B* = 179.61 (16)° in (II) and −177.5 (2)° in (III) and C2*B*—C3*B*—N3*B*—O32*B* = −166.31 (17)° in (II) and −155.2 (2)° in (III)]. Also, in (III), the phenolic substituent group (O2*B*) is disordered by rotation about the C1*B*⋯C4*B* ring vector giving a minor site-occupancy factor for the O21*B*—H21*B* group of 0.28 (SOF fixed in the final refinement cycles). This is similar to the disorder in three examples among the DNSA proton-transfer salts with Lewis bases, *e.g.* with nicotinamide (SOF = 0.76/0.24) (Koman *et al.*, 2003 ▸), with 2,6-di­amino­pyridine (0.90/0.10) (Smith *et al.*, 2003 ▸) and with quinoline-2-carb­oxy­lic acid (0.51/0.49) (Smith *et al.*, 2007 ▸). In (III), the usual short intra­molecular phenol O—H⋯O_carbox­yl_ hydrogen bond is present (Table 3 ▸).

## Supra­molecular features   

**Figure 4:**
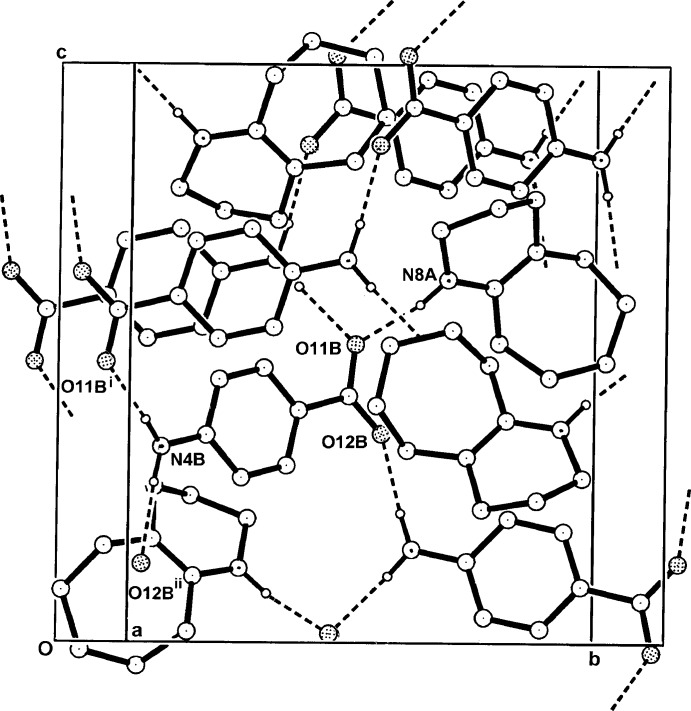
The three-dimensional hydrogen-bonded framework structure of (I) viewed approximately along *a*. For symmetry codes, see Table 1 ▸.

In the crystal of (I), the N8*A*—H⋯O11*B* hydrogen-bonded cation–anion pairs are extended through inter­molecular N4*B*—H⋯ O11*B* ^i^ and ⋯N12*B* ^ii^ hydrogen-bonding extensions (Table 1 ▸), giving an overall three-dimensional network structure (Fig. 4 ▸). The structure contains no inter-ring π–π inter­actions or C—H⋯O hydrogen bonds.

**Figure 5:**
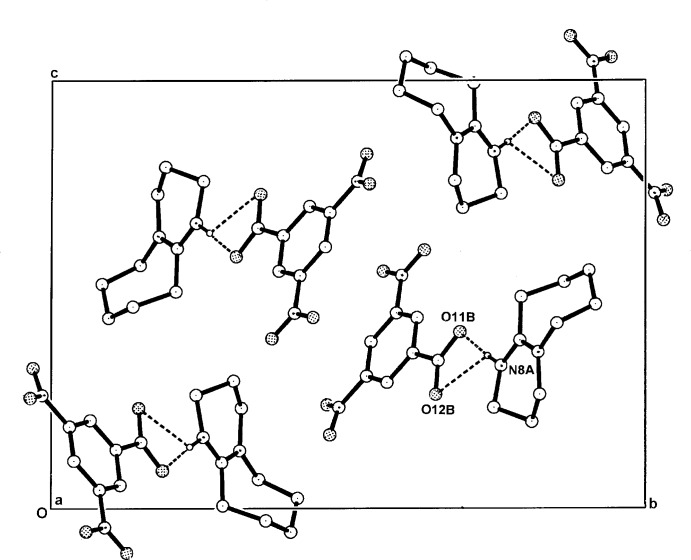
The packing of the hydrogen-bonded cation-anion pairs in the unit cell of (II), viewed along *a*. The minor-component disordered atoms and the non-associative H atoms have been omitted.

The unit-cell parameters, space group (Table 4 ▸), and the overall crystal packing of (II) and (III) are very similar (Figs. 5 ▸ and 6 ▸). Although no classical hydrogen-bonding inter­actions are present between the primary cation–anion pairs, with both structures there are two minor cation C—H⋯O hydrogen-bonding extensions to nitro O-atom acceptors, C2*A*—H⋯O31*B* ^ii^ [3.309 (2) Å in (II) and 3.281 (3) Å in (III)] and C10*A*—H⋯O32*B* ^i^ [3.247 (3) Å in (II) and 3.251 (5) Å in (III)] (Tables 2 ▸ and 3 ▸). These give two-dimensional layered structures lying parallel to (001). There are no inter-ring π–π inter­actions in either (II) or (III).

## Synthesis and crystallization   

The title compounds (I)–(III) were prepared by first dissolving 100 mg of either PABA, DNBA, or DNSA in 5 mL of warm ethanol followed by the addition, with stirring, of 111 mg (I), 72 mg (II) or 67 mg (III) of BDU, respectively. Slow evaporation at room temperature gave colourless needles of (I), colourless prisms of (II), and fine yellow needles of (III), from which specimens were cleaved for the X-ray analyses.

## Refinement details   

Crystal data, data collection and structure refinement details are given in Table 4 ▸. Hydrogen atoms were placed in calculated positions [C—H_aromatic_ = 0.95 Å or C—H_methyl­ene_ = 0.99 Å] and were allowed to ride in the refinements, with *U*_iso_(H) = 1.2*U*_eq_(C). The amine and aminium H-atoms were located in difference-Fourier analyses and were allowed to refine with distance restraints [N—H = 0.90 (2) Å] and with *U*_iso_(H) = 1.2*U*_eq_(N). Disorder involving atoms C9*A* and C10*A* of the six-membered ring systems of both (II) and (III) gave refined minor occupancy sites C12*A* and C13*A*, with site occupancy factors of 0.735 (3)/0.265 (3) and 0.686 (4)/0.314 (4), respectively. Also in (III), the phenol group of the DNSA anion was found to be disordered with the minor occupancy site (O21*B*) having a SOF = 0.28, which was fixed in the final cycles of refinement. In the structure of (I), although of no relevance in the achiral mol­ecule, the Flack parameter (Flack, 1983 ▸) was determined as −0.1 (13) for 1668 Friedel pairs, which serves to indicate the lack of any usable anomalous scattering signal, as expected for an all-light-atom structure determined with Mo *K*α X-rays.

## Supplementary Material

Crystal structure: contains datablock(s) global, I, II, III. DOI: 10.1107/S205698901600267X/pk2574sup1.cif

Structure factors: contains datablock(s) I. DOI: 10.1107/S205698901600267X/pk2574Isup2.hkl

Structure factors: contains datablock(s) II. DOI: 10.1107/S205698901600267X/pk2574IIsup3.hkl

Structure factors: contains datablock(s) III. DOI: 10.1107/S205698901600267X/pk2574IIIsup4.hkl

Supporting information file. DOI: 10.1107/S205698901600267X/pk2574Isup5.cml

Supporting information file. DOI: 10.1107/S205698901600267X/pk2574IIsup6.cml

Supporting information file. DOI: 10.1107/S205698901600267X/pk2574IIIsup7.cml

CCDC references: 1453494, 1453493, 1453492

Additional supporting information:  crystallographic information; 3D view; checkCIF report

## Figures and Tables

**Figure 6:**
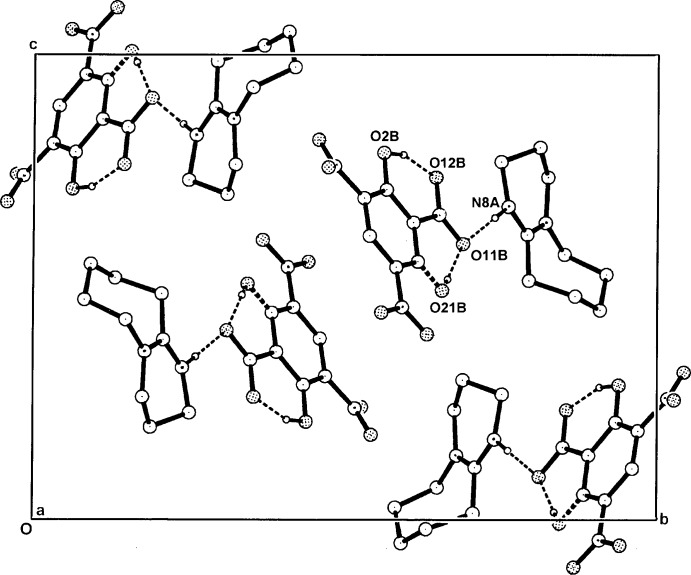
The packing of the hydrogen-bonded cation-anion pairs in the unit cell of (III), viewed along *a*. The minor-component disordered atoms and the non-associative H atoms have been omitted.

**Table 1:** Hydrogen-bond geometry (Å, °) for (I)

*D*—H⋯*A*	*D*—H	H⋯*A*	*D*⋯*A*	*D*—H⋯*A*
N8*A*—H8*A*⋯O11*B*	0.89 (2)	1.78 (2)	2.665 (2)	170 (2)
N4*B*—H41*B*⋯O11*B* ^i^	0.89 (2)	2.05 (2)	2.939 (2)	176 (2)
N4*B*—H42*B*⋯O12*B* ^ii^	0.92 (2)	1.98 (2)	2.891 (2)	176 (2)

Symmetry codes: (i) 
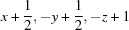
; (ii) 
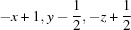
.

**Table 2:** Hydrogen-bond geometry (Å, °) for (II)

*D*—H⋯*A*	*D*—H	H⋯*A*	*D*⋯*A*	*D*—H⋯*A*
N8*A*—H8*A*⋯O11*B*	0.90 (2)	1.88 (2)	2.777 (2)	177 (2)
N8*A*—H8*A*⋯O12*B*	0.90 (2)	2.53 (2)	3.117 (2)	124 (1)
C10*A*—H11*A*⋯O32*B* ^i^	0.99	2.44	3.247 (3)	138
C2*A*—H21*A*⋯O31*B* ^ii^	0.99	2.56	3.309 (2)	133
C6*A*—H62*A*⋯O11*B*	0.99	2.60	3.438 (2)	143

Symmetry codes: (i) 
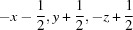
; (ii) 
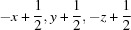
.

**Table 3:** Hydrogen-bond geometry (Å, °) for (III)

*D*—H⋯*A*	*D*—H	H⋯*A*	*D*⋯*A*	*D*—H⋯*A*
N8*A*—H8*A*⋯O11*B*	0.88 (2)	1.99 (2)	2.871 (3)	176 (2)
O2*B*—H2*B*⋯O12*B*	0.84	1.72	2.473 (3)	149
C10*A*—H11*A*⋯O32*B* ^i^	0.99	2.45	3.251 (5)	138
C2*A*—H21*A*⋯O31*B* ^ii^	0.99	2.48	3.281 (3)	138

Symmetry codes: (i) 
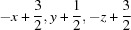
; (ii) 
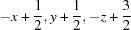
.

**Table 4:** Experimental details

	(I)	(II)	(III)
Crystal data			
Chemical formula	C_9_H_17_N_2_^+^·C_7_H_6_NO_2_^−^	C_9_H_17_N_2_^+^·C_7_H_3_N_2_O_6_^−^	C_9_H_17_N_2_^+^·C_7_H_3_N_2_O_7_^−^
*M*_r_	289.37	364.36	380.36
Crystal system, space group	Orthorhombic, *P*2_1_2_1_2_1_	Monoclinic, *P*2_1_/*n*	Monoclinic, *P*2_1_/*n*
Temperature (K)	200	200	200
*a*, *b*, *c* (Å)	8.0986 (4), 12.9213 (6), 13.7344 (7)	6.0197 (4), 19.6228 (13), 14.3866 (8)	6.1537 (3), 19.1541 (14), 14.5527 (11)
α, β, γ (°)	90, 90, 90	90, 98.078 (5), 90	90, 98.343 (6), 90
*V* (Å^3^)	1437.23 (12)	1682.53 (18)	1697.2 (2)
*Z*	4	4	4
Radiation type	Mo *K*α	Mo *K*α	Mo *K*α
μ (mm^−1^)	0.09	0.11	0.12
Crystal size (mm)	0.40 × 0.26 × 0.24	0.30 × 0.13 × 0.08	0.30 × 0.13 × 0.10

Data collection			
Diffractometer	Oxford Diffraction Gemini-S CCD-detector	Oxford Diffraction Gemini-S CCD-detector	Oxford Diffraction Gemini-S CCD-detector
Absorption correction	Multi-scan (*CrysAlis PRO*; Agilent, 2014 ▸)	Multi-scan (*CrysAlis PRO*; Agilent, 2014 ▸)	Multi-scan (*CrysAlis PRO*; Agilent, 2014 ▸)
*T*_min_, *T*_max_	0.93, 0.99	0.90, 0.99	0.920, 0.990
No. of measured, independent and observed [*I* > 2σ(*I*)] reflections	7372, 3324, 2847	7082, 3311, 2561	7800, 3339, 2347
*R*_int_	0.031	0.024	0.034
(sin θ/λ)_max_ (Å^−1^)	0.687	0.617	0.617

Refinement			
*R*[*F* ^2^ > 2σ(*F* ^2^)], *wR*(*F* ^2^), *S*	0.044, 0.098, 1.07	0.045, 0.109, 1.02	0.058, 0.123, 1.03
No. of reflections	3324	3311	3339
No. of parameters	199	245	263
No. of restraints	3	3	3
H-atom treatment	H atoms treated by a mixture of independent and constrained refinement	H atoms treated by a mixture of independent and constrained refinement	H atoms treated by a mixture of independent and constrained refinement
Δρ_max_, Δρ_min_ (e Å^−3^)	0.20, −0.25	0.18, −0.22	0.29, −0.29

Computer programs: *CrysAlis PRO* (Agilent, 2014 ▸), *SIR92* (Altomare *et al.*, 1993 ▸), *SHELXL97* (Sheldrick, 2008 ▸) within *WinGX* (Farrugia, 2012 ▸) and *PLATON* (Spek, 2009 ▸).

## supplementary crystallographic information

### (I) 1-Aza-8-azoniabicyclo[5.4.0]undec-7-ene 4-aminobenzoate Crystal data

**Table:**

C_9_H_17_N_2_^+^·C_7_H_6_NO_2_^−^	*F*(000) = 624
*M**_r_* = 289.37	*D*_x_ = 1.337 Mg m^−^^3^
Orthorhombic, *P*2_1_2_1_2_1_	Mo *K*α radiation, λ = 0.71073 Å
Hall symbol: P 2ac 2ab	Cell parameters from 2097 reflections
*a* = 8.0986 (4) Å	θ = 3.5–28.4°
*b* = 12.9213 (6) Å	µ = 0.09 mm^−^^1^
*c* = 13.7344 (7) Å	*T* = 200 K
*V* = 1437.23 (12) Å^3^	Prism, colourless
*Z* = 4	0.40 × 0.26 × 0.24 mm

### (I) 1-Aza-8-azoniabicyclo[5.4.0]undec-7-ene 4-aminobenzoate Data collection

**Table:**

Oxford Diffraction Gemini-S CCD-detector diffractometer	3324 independent reflections
Radiation source: Enhance (Mo) X-ray source	2847 reflections with *I* > 2σ(*I*)
Graphite monochromator	*R*_int_ = 0.031
Detector resolution: 16.067 pixels mm^-1^	θ_max_ = 29.2°, θ_min_ = 3.3°
ω scans	*h* = −10→10
Absorption correction: multi-scan (*CrysAlis PRO*; Agilent, 2014)	*k* = −16→15
*T*_min_ = 0.93, *T*_max_ = 0.99	*l* = −17→18
7372 measured reflections	

### (I) 1-Aza-8-azoniabicyclo[5.4.0]undec-7-ene 4-aminobenzoate Refinement

**Table:**

Refinement on *F*^2^	Secondary atom site location: difference Fourier map
Least-squares matrix: full	Hydrogen site location: inferred from neighbouring sites
*R*[*F*^2^ > 2σ(*F*^2^)] = 0.044	H atoms treated by a mixture of independent and constrained refinement
*wR*(*F*^2^) = 0.098	*w* = 1/[σ^2^(*F*_o_^2^) + (0.0438*P*)^2^ + 0.0476*P*] where *P* = (*F*_o_^2^ + 2*F*_c_^2^)/3
*S* = 1.07	(Δ/σ)_max_ < 0.001
3324 reflections	Δρ_max_ = 0.20 e Å^−^^3^
199 parameters	Δρ_min_ = −0.25 e Å^−^^3^
3 restraints	Absolute structure: Flack (1983), 1668 Friedel pairs
Primary atom site location: structure-invariant direct methods	Absolute structure parameter: −0.1 (13)

### (I) 1-Aza-8-azoniabicyclo[5.4.0]undec-7-ene 4-aminobenzoate Special details

**Table:**

Geometry. Bond distances, angles etc. have been calculated using the rounded fractional coordinates. All su's are estimated from the variances of the (full) variance-covariance matrix. The cell esds are taken into account in the estimation of distances, angles and torsion angles
Refinement. Refinement of F^2^ against ALL reflections. The weighted R-factor wR and goodness of fit S are based on F^2^, conventional R-factors R are based on F, with F set to zero for negative F^2^. The threshold expression of F^2^ > 2sigma(F^2^) is used only for calculating R-factors(gt) etc. and is not relevant to the choice of reflections for refinement. R-factors based on F^2^ are statistically about twice as large as those based on F, and R- factors based on ALL data will be even larger.

### (I) 1-Aza-8-azoniabicyclo[5.4.0]undec-7-ene 4-aminobenzoate Fractional atomic coordinates and isotropic or equivalent isotropic displacement parameters (Å^2^)

**Table:**

	*x*	*y*	*z*	*U*_iso_*/*U*_eq_	
N1A	0.32105 (18)	0.84571 (11)	0.67893 (11)	0.0229 (4)	
N8A	0.36282 (18)	0.67732 (12)	0.62864 (11)	0.0241 (5)	
C2A	0.2390 (2)	0.94651 (14)	0.66676 (13)	0.0256 (5)	
C3A	0.3174 (2)	1.01454 (14)	0.58999 (14)	0.0291 (6)	
C4A	0.2728 (2)	0.98456 (14)	0.48576 (14)	0.0288 (6)	
C5A	0.3145 (2)	0.87339 (14)	0.45932 (13)	0.0271 (5)	
C6A	0.2207 (2)	0.79201 (14)	0.51882 (13)	0.0262 (5)	
C7A	0.3028 (2)	0.77086 (13)	0.61456 (13)	0.0209 (5)	
C9A	0.4591 (2)	0.64922 (14)	0.71447 (13)	0.0262 (5)	
C10A	0.5429 (2)	0.74497 (13)	0.75333 (13)	0.0280 (6)	
C11A	0.4170 (2)	0.82988 (15)	0.76868 (13)	0.0302 (6)	
O11B	0.28719 (17)	0.51621 (9)	0.51597 (9)	0.0320 (4)	
O12B	0.29529 (19)	0.56473 (11)	0.36120 (11)	0.0428 (5)	
N4B	0.6170 (2)	0.11141 (13)	0.33808 (12)	0.0296 (5)	
C1B	0.3958 (2)	0.39741 (13)	0.40190 (12)	0.0206 (5)	
C2B	0.43611 (19)	0.36990 (13)	0.30648 (12)	0.0212 (5)	
C3B	0.5089 (2)	0.27615 (13)	0.28504 (12)	0.0220 (5)	
C4B	0.5475 (2)	0.20495 (13)	0.35867 (13)	0.0220 (5)	
C5B	0.5100 (2)	0.23325 (13)	0.45489 (12)	0.0243 (5)	
C6B	0.4347 (2)	0.32664 (13)	0.47496 (13)	0.0227 (5)	
C11B	0.3204 (2)	0.50006 (14)	0.42672 (14)	0.0238 (5)	
H8A	0.342 (2)	0.6279 (14)	0.5850 (13)	0.0290*	
H10A	0.59810	0.72880	0.81580	0.0340*	
H11A	0.34180	0.81070	0.82260	0.0360*	
H12A	0.47390	0.89490	0.78670	0.0360*	
H13A	0.62810	0.76860	0.70660	0.0340*	
H21A	0.24080	0.98360	0.72980	0.0310*	
H22A	0.12200	0.93460	0.64920	0.0310*	
H31A	0.43890	1.01140	0.59740	0.0350*	
H32A	0.28290	1.08700	0.60140	0.0350*	
H41A	0.15290	0.99540	0.47620	0.0350*	
H42A	0.33170	1.03140	0.44050	0.0350*	
H51A	0.43450	0.86260	0.46870	0.0320*	
H52A	0.29000	0.86260	0.38940	0.0320*	
H61A	0.10660	0.81660	0.53060	0.0310*	
H62A	0.21410	0.72690	0.48100	0.0310*	
H91A	0.54280	0.59660	0.69700	0.0310*	
H92A	0.38570	0.61950	0.76490	0.0310*	
H2B	0.41290	0.41690	0.25510	0.0250*	
H3B	0.53320	0.25950	0.21920	0.0260*	
H5B	0.53680	0.18760	0.50670	0.0290*	
H6B	0.40870	0.34320	0.54060	0.0270*	
H41B	0.666 (2)	0.0742 (16)	0.3845 (13)	0.0360*	
H42B	0.647 (2)	0.0939 (16)	0.2759 (12)	0.0360*	

### (I) 1-Aza-8-azoniabicyclo[5.4.0]undec-7-ene 4-aminobenzoate Atomic displacement parameters (Å^2^)

**Table:**

	*U*^11^	*U*^22^	*U*^33^	*U*^12^	*U*^13^	*U*^23^
N1A	0.0263 (7)	0.0220 (8)	0.0205 (7)	0.0030 (6)	−0.0033 (6)	−0.0022 (6)
N8A	0.0294 (8)	0.0197 (8)	0.0233 (8)	−0.0004 (6)	−0.0008 (6)	−0.0024 (7)
C2A	0.0276 (9)	0.0223 (9)	0.0269 (10)	0.0053 (7)	−0.0018 (7)	−0.0053 (8)
C3A	0.0311 (10)	0.0218 (9)	0.0343 (11)	−0.0005 (8)	−0.0031 (8)	−0.0024 (8)
C4A	0.0314 (10)	0.0263 (9)	0.0287 (10)	0.0011 (8)	−0.0008 (8)	0.0041 (8)
C5A	0.0295 (9)	0.0292 (10)	0.0225 (9)	0.0039 (8)	−0.0010 (7)	−0.0007 (8)
C6A	0.0312 (9)	0.0221 (9)	0.0253 (9)	−0.0010 (7)	−0.0062 (8)	−0.0035 (8)
C7A	0.0202 (8)	0.0203 (9)	0.0223 (9)	−0.0013 (7)	0.0013 (7)	−0.0012 (7)
C9A	0.0268 (9)	0.0251 (9)	0.0267 (10)	0.0030 (8)	−0.0003 (7)	0.0033 (8)
C10A	0.0269 (9)	0.0301 (10)	0.0269 (10)	0.0034 (8)	−0.0065 (8)	−0.0010 (8)
C11A	0.0365 (11)	0.0306 (10)	0.0235 (9)	0.0053 (8)	−0.0094 (8)	−0.0056 (8)
O11B	0.0520 (8)	0.0207 (7)	0.0233 (7)	−0.0025 (6)	0.0074 (6)	−0.0035 (5)
O12B	0.0643 (9)	0.0337 (8)	0.0305 (8)	0.0187 (7)	0.0123 (7)	0.0099 (7)
N4B	0.0406 (9)	0.0260 (9)	0.0223 (9)	0.0072 (7)	0.0000 (7)	−0.0008 (7)
C1B	0.0194 (8)	0.0215 (9)	0.0209 (9)	−0.0045 (6)	−0.0004 (7)	0.0005 (7)
C2B	0.0225 (9)	0.0237 (9)	0.0174 (8)	−0.0015 (7)	−0.0013 (6)	0.0033 (7)
C3B	0.0240 (8)	0.0241 (8)	0.0179 (8)	−0.0032 (7)	0.0001 (7)	0.0010 (7)
C4B	0.0216 (8)	0.0195 (9)	0.0250 (9)	−0.0025 (7)	−0.0011 (7)	−0.0019 (7)
C5B	0.0322 (9)	0.0221 (9)	0.0185 (8)	0.0004 (8)	−0.0011 (7)	0.0046 (7)
C6B	0.0288 (9)	0.0228 (9)	0.0166 (8)	−0.0043 (7)	0.0018 (7)	−0.0017 (7)
C11B	0.0255 (9)	0.0221 (9)	0.0237 (9)	−0.0043 (7)	0.0028 (7)	0.0001 (7)

### (I) 1-Aza-8-azoniabicyclo[5.4.0]undec-7-ene 4-aminobenzoate Geometric parameters (Å, º)

**Table:**

O11B—C11B	1.272 (2)	C4A—H41A	0.9900
O12B—C11B	1.245 (2)	C5A—H52A	0.9900
N1A—C11A	1.471 (2)	C5A—H51A	0.9900
N1A—C7A	1.319 (2)	C6A—H61A	0.9900
N1A—C2A	1.472 (2)	C6A—H62A	0.9900
N8A—C7A	1.317 (2)	C9A—H91A	0.9900
N8A—C9A	1.459 (2)	C9A—H92A	0.9900
N8A—H8A	0.892 (18)	C10A—H10A	0.9900
N4B—C4B	1.363 (2)	C10A—H13A	0.9900
N4B—H41B	0.892 (18)	C11A—H12A	0.9900
N4B—H42B	0.916 (17)	C11A—H11A	0.9900
C2A—C3A	1.513 (3)	C1B—C11B	1.499 (2)
C3A—C4A	1.526 (3)	C1B—C2B	1.397 (2)
C4A—C5A	1.520 (3)	C1B—C6B	1.394 (2)
C5A—C6A	1.533 (2)	C2B—C3B	1.379 (2)
C6A—C7A	1.499 (2)	C3B—C4B	1.402 (2)
C9A—C10A	1.509 (2)	C4B—C5B	1.404 (2)
C10A—C11A	1.513 (2)	C5B—C6B	1.380 (2)
C2A—H21A	0.9900	C2B—H2B	0.9500
C2A—H22A	0.9900	C3B—H3B	0.9500
C3A—H31A	0.9900	C5B—H5B	0.9500
C3A—H32A	0.9900	C6B—H6B	0.9500
C4A—H42A	0.9900		
			
C2A—N1A—C7A	121.49 (15)	C5A—C6A—H62A	109.00
C2A—N1A—C11A	117.17 (14)	H61A—C6A—H62A	108.00
C7A—N1A—C11A	121.26 (15)	C7A—C6A—H62A	109.00
C7A—N8A—C9A	122.97 (15)	C7A—C6A—H61A	109.00
C7A—N8A—H8A	119.3 (11)	C10A—C9A—H91A	110.00
C9A—N8A—H8A	117.8 (12)	N8A—C9A—H92A	110.00
C4B—N4B—H41B	120.9 (13)	N8A—C9A—H91A	110.00
H41B—N4B—H42B	114.5 (17)	C10A—C9A—H92A	110.00
C4B—N4B—H42B	121.5 (13)	H91A—C9A—H92A	108.00
N1A—C2A—C3A	113.83 (14)	C9A—C10A—H10A	110.00
C2A—C3A—C4A	114.02 (15)	H10A—C10A—H13A	108.00
C3A—C4A—C5A	114.29 (15)	C9A—C10A—H13A	110.00
C4A—C5A—C6A	114.26 (14)	C11A—C10A—H10A	110.00
C5A—C6A—C7A	111.90 (14)	C11A—C10A—H13A	110.00
N8A—C7A—C6A	117.38 (15)	N1A—C11A—H11A	110.00
N1A—C7A—N8A	122.23 (16)	N1A—C11A—H12A	110.00
N1A—C7A—C6A	120.28 (15)	C10A—C11A—H11A	110.00
N8A—C9A—C10A	108.79 (14)	H11A—C11A—H12A	108.00
C9A—C10A—C11A	109.93 (14)	C10A—C11A—H12A	110.00
N1A—C11A—C10A	109.88 (14)	C6B—C1B—C11B	120.58 (15)
C3A—C2A—H22A	109.00	C2B—C1B—C11B	122.25 (15)
H21A—C2A—H22A	108.00	C2B—C1B—C6B	117.12 (15)
N1A—C2A—H22A	109.00	C1B—C2B—C3B	121.59 (15)
C3A—C2A—H21A	109.00	C2B—C3B—C4B	121.17 (15)
N1A—C2A—H21A	109.00	N4B—C4B—C3B	121.61 (16)
C2A—C3A—H31A	109.00	N4B—C4B—C5B	121.03 (16)
C2A—C3A—H32A	109.00	C3B—C4B—C5B	117.36 (15)
H31A—C3A—H32A	108.00	C4B—C5B—C6B	120.75 (16)
C4A—C3A—H31A	109.00	C1B—C6B—C5B	122.00 (16)
C4A—C3A—H32A	109.00	O11B—C11B—O12B	123.50 (17)
C3A—C4A—H42A	109.00	O11B—C11B—C1B	116.76 (16)
H41A—C4A—H42A	108.00	O12B—C11B—C1B	119.74 (17)
C5A—C4A—H41A	109.00	C1B—C2B—H2B	119.00
C5A—C4A—H42A	109.00	C3B—C2B—H2B	119.00
C3A—C4A—H41A	109.00	C2B—C3B—H3B	119.00
C6A—C5A—H52A	109.00	C4B—C3B—H3B	119.00
H51A—C5A—H52A	108.00	C4B—C5B—H5B	120.00
C4A—C5A—H52A	109.00	C6B—C5B—H5B	120.00
C6A—C5A—H51A	109.00	C1B—C6B—H6B	119.00
C4A—C5A—H51A	109.00	C5B—C6B—H6B	119.00
C5A—C6A—H61A	109.00		
			
C7A—N1A—C2A—C3A	−74.8 (2)	N8A—C9A—C10A—C11A	52.82 (18)
C11A—N1A—C2A—C3A	108.53 (17)	C9A—C10A—C11A—N1A	−52.69 (19)
C2A—N1A—C7A—N8A	−173.67 (15)	C6B—C1B—C2B—C3B	1.0 (2)
C2A—N1A—C7A—C6A	10.2 (2)	C11B—C1B—C2B—C3B	178.48 (15)
C11A—N1A—C7A—N8A	2.9 (3)	C2B—C1B—C6B—C5B	0.1 (2)
C11A—N1A—C7A—C6A	−173.23 (15)	C11B—C1B—C6B—C5B	−177.44 (15)
C2A—N1A—C11A—C10A	−157.91 (14)	C2B—C1B—C11B—O11B	179.25 (15)
C7A—N1A—C11A—C10A	25.4 (2)	C2B—C1B—C11B—O12B	−1.6 (3)
C9A—N8A—C7A—N1A	−2.2 (3)	C6B—C1B—C11B—O11B	−3.4 (2)
C9A—N8A—C7A—C6A	174.06 (15)	C6B—C1B—C11B—O12B	175.83 (16)
C7A—N8A—C9A—C10A	−26.7 (2)	C1B—C2B—C3B—C4B	−0.9 (2)
N1A—C2A—C3A—C4A	77.87 (18)	C2B—C3B—C4B—N4B	178.92 (16)
C2A—C3A—C4A—C5A	−56.71 (19)	C2B—C3B—C4B—C5B	−0.4 (2)
C3A—C4A—C5A—C6A	62.97 (19)	N4B—C4B—C5B—C6B	−177.86 (16)
C4A—C5A—C6A—C7A	−83.76 (18)	C3B—C4B—C5B—C6B	1.4 (2)
C5A—C6A—C7A—N1A	60.9 (2)	C4B—C5B—C6B—C1B	−1.3 (3)
C5A—C6A—C7A—N8A	−115.39 (17)		

### (I) 1-Aza-8-azoniabicyclo[5.4.0]undec-7-ene 4-aminobenzoate Hydrogen-bond geometry (Å, º)

**Table:**

*D*—H···*A*	*D*—H	H···*A*	*D*···*A*	*D*—H···*A*
N8*A*—H8*A*···O11*B*	0.89 (2)	1.78 (2)	2.665 (2)	170 (2)
N4*B*—H41*B*···O11*B*^i^	0.89 (2)	2.05 (2)	2.939 (2)	176 (2)
N4*B*—H42*B*···O12*B*^ii^	0.92 (2)	1.98 (2)	2.891 (2)	176 (2)

Symmetry codes: (i) *x*+1/2, −*y*+1/2, −*z*+1; (ii) −*x*+1, *y*−1/2, −*z*+1/2.

### (II) Aza-8-azoniabicyclo[5.4.0]undec-7-ene 3,5-dinitrobenzoate Crystal data

**Table:**

C_9_H_17_N_2_^+^·C_7_H_3_N_2_O_6_^−^	*F*(000) = 768
*M**_r_* = 364.36	*D*_x_ = 1.438 Mg m^−^^3^
Monoclinic, *P*2_1_/*n*	Mo *K*α radiation, λ = 0.71073 Å
Hall symbol: -P 2yn	Cell parameters from 1784 reflections
*a* = 6.0197 (4) Å	θ = 4.0–28.0°
*b* = 19.6228 (13) Å	µ = 0.11 mm^−^^1^
*c* = 14.3866 (8) Å	*T* = 200 K
β = 98.078 (5)°	Needle, colourless
*V* = 1682.53 (18) Å^3^	0.30 × 0.13 × 0.08 mm
*Z* = 4	

### (II) Aza-8-azoniabicyclo[5.4.0]undec-7-ene 3,5-dinitrobenzoate Data collection

**Table:**

Oxford Diffraction Gemini-S CCD-detector diffractometer	3311 independent reflections
Radiation source: fine-focus sealed tube	2561 reflections with *I* > 2σ(*I*)
Graphite monochromator	*R*_int_ = 0.024
Detector resolution: 16.077 pixels mm^-1^	θ_max_ = 26.0°, θ_min_ = 3.4°
ω scans	*h* = −7→7
Absorption correction: multi-scan (*CrysAlis PRO*; Agilent, 2014)	*k* = −14→24
*T*_min_ = 0.90, *T*_max_ = 0.99	*l* = −9→17
7082 measured reflections	

### (II) Aza-8-azoniabicyclo[5.4.0]undec-7-ene 3,5-dinitrobenzoate Refinement

**Table:**

Refinement on *F*^2^	Primary atom site location: structure-invariant direct methods
Least-squares matrix: full	Secondary atom site location: difference Fourier map
*R*[*F*^2^ > 2σ(*F*^2^)] = 0.045	Hydrogen site location: inferred from neighbouring sites
*wR*(*F*^2^) = 0.109	H atoms treated by a mixture of independent and constrained refinement
*S* = 1.01	*w* = 1/[σ^2^(*F*_o_^2^) + (0.0435*P*)^2^ + 0.5615*P*] where *P* = (*F*_o_^2^ + 2*F*_c_^2^)/3
3311 reflections	(Δ/σ)_max_ < 0.001
245 parameters	Δρ_max_ = 0.18 e Å^−^^3^
3 restraints	Δρ_min_ = −0.22 e Å^−^^3^

### (II) Aza-8-azoniabicyclo[5.4.0]undec-7-ene 3,5-dinitrobenzoate Special details

**Table:**

Geometry. Bond distances, angles etc. have been calculated using the rounded fractional coordinates. All su's are estimated from the variances of the (full) variance-covariance matrix. The cell esds are taken into account in the estimation of distances, angles and torsion angles
Refinement. Refinement of F^2^ against ALL reflections. The weighted R-factor wR and goodness of fit S are based on F^2^, conventional R-factors R are based on F, with F set to zero for negative F^2^. The threshold expression of F^2^ > 2sigma(F^2^) is used only for calculating R-factors(gt) etc. and is not relevant to the choice of reflections for refinement. R-factors based on F^2^ are statistically about twice as large as those based on F, and R- factors based on ALL data will be even larger.

### (II) Aza-8-azoniabicyclo[5.4.0]undec-7-ene 3,5-dinitrobenzoate Fractional atomic coordinates and isotropic or equivalent isotropic displacement parameters (Å^2^)

**Table:**

	*x*	*y*	*z*	*U*_iso_*/*U*_eq_	Occ. (<1)
O11B	−0.0061 (2)	0.68797 (7)	0.41185 (9)	0.0408 (4)	
O12B	−0.0380 (2)	0.64765 (8)	0.26602 (10)	0.0556 (5)	
O31B	−0.5921 (3)	0.46963 (8)	0.17213 (10)	0.0567 (5)	
O32B	−0.8865 (3)	0.46500 (9)	0.24178 (11)	0.0703 (6)	
O51B	−0.8471 (2)	0.55899 (8)	0.55381 (10)	0.0514 (5)	
O52B	−0.5787 (3)	0.62813 (8)	0.60351 (10)	0.0576 (6)	
N3B	−0.6966 (3)	0.48464 (8)	0.23584 (11)	0.0416 (5)	
N5B	−0.6770 (3)	0.59011 (8)	0.54409 (10)	0.0363 (5)	
C1B	−0.2967 (3)	0.60944 (8)	0.36270 (11)	0.0264 (5)	
C2B	−0.3972 (3)	0.56537 (8)	0.29419 (11)	0.0288 (5)	
C3B	−0.5892 (3)	0.53100 (8)	0.30888 (11)	0.0289 (5)	
C4B	−0.6880 (3)	0.53844 (8)	0.38905 (12)	0.0293 (5)	
C5B	−0.5807 (3)	0.58130 (8)	0.45649 (11)	0.0265 (5)	
C6B	−0.3873 (3)	0.61637 (8)	0.44556 (11)	0.0269 (5)	
C11B	−0.0952 (3)	0.65174 (9)	0.34516 (13)	0.0327 (5)	
N1A	0.6514 (2)	0.81846 (7)	0.36517 (9)	0.0288 (4)	
N8A	0.3270 (3)	0.75625 (8)	0.33371 (10)	0.0364 (5)	
C2A	0.8281 (3)	0.85009 (9)	0.43221 (13)	0.0350 (6)	
C3A	0.7557 (3)	0.91508 (9)	0.47621 (13)	0.0378 (6)	
C4A	0.6172 (3)	0.90383 (10)	0.55531 (13)	0.0390 (6)	
C5A	0.4046 (3)	0.86226 (10)	0.52884 (13)	0.0383 (6)	
C6A	0.4433 (3)	0.78996 (9)	0.49381 (11)	0.0334 (5)	
C7A	0.4773 (3)	0.78797 (8)	0.39270 (11)	0.0262 (5)	
C9A	0.3565 (6)	0.74500 (17)	0.2353 (2)	0.0357 (10)	0.735 (3)
C10A	0.4681 (5)	0.80757 (15)	0.20241 (17)	0.0364 (8)	0.735 (3)
C11A	0.6839 (3)	0.82115 (10)	0.26593 (12)	0.0364 (6)	
C13A	0.3000 (16)	0.7705 (5)	0.2305 (8)	0.0357 (10)	0.265 (3)
C12A	0.5368 (12)	0.7669 (4)	0.2074 (5)	0.0364 (8)	0.265 (3)
H2B	−0.33470	0.55890	0.23780	0.0350*	
H4B	−0.82260	0.51530	0.39720	0.0350*	
H6B	−0.31700	0.64500	0.49430	0.0320*	
H8A	0.217 (3)	0.7342 (9)	0.3570 (12)	0.0440*	
H10A	0.49920	0.80070	0.13730	0.0440*	0.735 (3)
H21A	0.95830	0.86030	0.39950	0.0420*	
H22A	0.87810	0.81700	0.48270	0.0420*	
H31A	0.66700	0.94280	0.42680	0.0450*	
H32A	0.89130	0.94160	0.50080	0.0450*	
H41A	0.57520	0.94880	0.57870	0.0470*	
H42A	0.71200	0.88050	0.60760	0.0470*	
H51A	0.30550	0.88690	0.47920	0.0460*	
H52A	0.32480	0.85900	0.58440	0.0460*	
H61A	0.57690	0.77030	0.53250	0.0400*	
H62A	0.31250	0.76120	0.50230	0.0400*	
H91A	0.20920	0.73760	0.19650	0.0430*	0.735 (3)
H92A	0.45120	0.70430	0.22990	0.0430*	0.735 (3)
H11A	0.36680	0.84730	0.20280	0.0440*	0.735 (3)
H12A	0.79710	0.78680	0.25410	0.0440*	0.735 (3)
H13A	0.74150	0.86670	0.25170	0.0440*	0.735 (3)
H14A	0.53670	0.77550	0.13960	0.0440*	0.265 (3)
H15A	0.59940	0.72090	0.22210	0.0440*	0.265 (3)
H16A	0.23430	0.81620	0.21620	0.0430*	0.265 (3)
H17A	0.20330	0.73590	0.19490	0.0430*	0.265 (3)
H18A	0.84390	0.81290	0.26060	0.0440*	0.265 (3)
H19A	0.64380	0.86710	0.24050	0.0440*	0.265 (3)

### (II) Aza-8-azoniabicyclo[5.4.0]undec-7-ene 3,5-dinitrobenzoate Atomic displacement parameters (Å^2^)

**Table:**

	*U*^11^	*U*^22^	*U*^33^	*U*^12^	*U*^13^	*U*^23^
O11B	0.0376 (7)	0.0403 (8)	0.0454 (7)	−0.0139 (6)	0.0090 (6)	−0.0054 (6)
O12B	0.0558 (9)	0.0740 (11)	0.0419 (8)	−0.0258 (8)	0.0238 (7)	−0.0043 (7)
O31B	0.0690 (10)	0.0566 (10)	0.0423 (8)	0.0063 (8)	0.0003 (7)	−0.0213 (7)
O32B	0.0733 (11)	0.0811 (12)	0.0539 (9)	−0.0503 (10)	−0.0006 (8)	−0.0091 (9)
O51B	0.0472 (8)	0.0586 (9)	0.0543 (8)	−0.0128 (7)	0.0276 (7)	0.0036 (7)
O52B	0.0663 (10)	0.0729 (11)	0.0380 (8)	−0.0175 (8)	0.0228 (7)	−0.0229 (8)
N3B	0.0534 (11)	0.0353 (9)	0.0329 (8)	−0.0074 (8)	−0.0055 (8)	−0.0016 (7)
N5B	0.0392 (9)	0.0378 (9)	0.0343 (8)	−0.0006 (7)	0.0132 (7)	0.0021 (7)
C1B	0.0250 (8)	0.0242 (8)	0.0298 (8)	0.0011 (7)	0.0034 (7)	0.0032 (7)
C2B	0.0342 (9)	0.0280 (9)	0.0251 (8)	0.0037 (7)	0.0070 (7)	0.0017 (7)
C3B	0.0338 (9)	0.0244 (9)	0.0268 (8)	−0.0015 (7)	−0.0021 (7)	−0.0001 (7)
C4B	0.0263 (8)	0.0258 (9)	0.0352 (9)	−0.0016 (7)	0.0021 (7)	0.0055 (8)
C5B	0.0284 (8)	0.0259 (9)	0.0264 (8)	0.0035 (7)	0.0078 (7)	0.0013 (7)
C6B	0.0275 (8)	0.0247 (9)	0.0279 (8)	−0.0004 (7)	0.0020 (7)	−0.0018 (7)
C11B	0.0297 (9)	0.0309 (9)	0.0382 (10)	−0.0006 (8)	0.0072 (8)	0.0031 (8)
N1A	0.0255 (7)	0.0325 (8)	0.0285 (7)	−0.0044 (6)	0.0039 (6)	0.0020 (6)
N8A	0.0358 (8)	0.0484 (10)	0.0250 (7)	−0.0178 (7)	0.0045 (6)	−0.0008 (7)
C2A	0.0236 (9)	0.0388 (10)	0.0410 (10)	−0.0059 (8)	−0.0007 (8)	0.0001 (8)
C3A	0.0347 (10)	0.0330 (10)	0.0434 (10)	−0.0076 (8)	−0.0020 (8)	0.0000 (9)
C4A	0.0405 (10)	0.0399 (11)	0.0343 (9)	−0.0025 (8)	−0.0032 (8)	−0.0064 (9)
C5A	0.0357 (10)	0.0495 (12)	0.0302 (9)	−0.0046 (9)	0.0062 (8)	−0.0083 (8)
C6A	0.0357 (10)	0.0403 (10)	0.0237 (8)	−0.0113 (8)	0.0024 (7)	0.0050 (8)
C7A	0.0254 (8)	0.0250 (8)	0.0272 (8)	−0.0015 (7)	0.0006 (7)	0.0028 (7)
C9A	0.0418 (19)	0.041 (2)	0.0236 (10)	−0.0039 (14)	0.0026 (12)	−0.0018 (16)
C10A	0.0443 (15)	0.0390 (16)	0.0263 (10)	−0.0024 (12)	0.0061 (10)	0.0036 (12)
C11A	0.0348 (10)	0.0427 (11)	0.0339 (9)	−0.0043 (8)	0.0126 (8)	0.0047 (8)
C13A	0.0418 (19)	0.041 (2)	0.0236 (10)	−0.0039 (14)	0.0026 (12)	−0.0018 (16)
C12A	0.0443 (15)	0.0390 (16)	0.0263 (10)	−0.0024 (12)	0.0061 (10)	0.0036 (12)

### (II) Aza-8-azoniabicyclo[5.4.0]undec-7-ene 3,5-dinitrobenzoate Geometric parameters (Å, º)

**Table:**

O11B—C11B	1.253 (2)	C5A—C6A	1.534 (3)
O12B—C11B	1.238 (2)	C6A—C7A	1.498 (2)
O31B—N3B	1.218 (2)	C9A—C10A	1.507 (4)
O32B—N3B	1.221 (3)	C10A—C11A	1.503 (3)
O51B—N5B	1.217 (2)	C12A—C13A	1.510 (12)
O52B—N5B	1.223 (2)	C12A—C11A	1.555 (8)
N3B—C3B	1.469 (2)	C2A—H21A	0.9900
N5B—C5B	1.470 (2)	C2A—H22A	0.9900
N1A—C2A	1.469 (2)	C3A—H31A	0.9900
N1A—C7A	1.315 (2)	C3A—H32A	0.9900
N1A—C11A	1.469 (2)	C4A—H41A	0.9900
N8A—C13A	1.497 (11)	C4A—H42A	0.9900
N8A—C9A	1.468 (3)	C5A—H51A	0.9900
N8A—C7A	1.308 (2)	C5A—H52A	0.9900
N8A—H8A	0.895 (18)	C6A—H61A	0.9900
C1B—C11B	1.520 (3)	C6A—H62A	0.9900
C1B—C2B	1.385 (2)	C9A—H91A	0.9900
C1B—C6B	1.386 (2)	C9A—H92A	0.9900
C2B—C3B	1.380 (2)	C10A—H10A	0.9900
C3B—C4B	1.378 (2)	C10A—H11A	0.9900
C4B—C5B	1.375 (2)	C11A—H12A	0.9900
C5B—C6B	1.380 (2)	C11A—H13A	0.9900
C2B—H2B	0.9500	C12A—H14A	0.9900
C4B—H4B	0.9500	C12A—H15A	0.9900
C6B—H6B	0.9500	C13A—H16A	0.9900
C2A—C3A	1.515 (3)	C13A—H17A	0.9900
C3A—C4A	1.518 (3)	C11A—H18A	0.9900
C4A—C5A	1.520 (3)	C11A—H19A	0.9900
			
O31B—N3B—O32B	124.33 (17)	C3A—C2A—H22A	109.00
O31B—N3B—C3B	117.77 (17)	H21A—C2A—H22A	108.00
O32B—N3B—C3B	117.89 (16)	C2A—C3A—H31A	109.00
O51B—N5B—O52B	123.92 (16)	C2A—C3A—H32A	109.00
O51B—N5B—C5B	118.63 (15)	C4A—C3A—H31A	109.00
O52B—N5B—C5B	117.44 (16)	C4A—C3A—H32A	109.00
C2A—N1A—C11A	116.09 (13)	H31A—C3A—H32A	108.00
C7A—N1A—C11A	121.98 (14)	C3A—C4A—H41A	109.00
C2A—N1A—C7A	121.91 (14)	C3A—C4A—H42A	109.00
C7A—N8A—C9A	122.1 (2)	C5A—C4A—H41A	108.00
C7A—N8A—C13A	121.6 (4)	C5A—C4A—H42A	108.00
C13A—N8A—H8A	118.6 (12)	H41A—C4A—H42A	108.00
C9A—N8A—H8A	119.0 (11)	C4A—C5A—H51A	109.00
C7A—N8A—H8A	117.9 (11)	C4A—C5A—H52A	109.00
C2B—C1B—C6B	119.23 (16)	C6A—C5A—H51A	109.00
C2B—C1B—C11B	120.13 (15)	C6A—C5A—H52A	109.00
C6B—C1B—C11B	120.60 (15)	H51A—C5A—H52A	108.00
C1B—C2B—C3B	119.19 (15)	C5A—C6A—H61A	109.00
N3B—C3B—C2B	119.14 (15)	C5A—C6A—H62A	109.00
N3B—C3B—C4B	117.78 (16)	C7A—C6A—H61A	109.00
C2B—C3B—C4B	123.08 (15)	C7A—C6A—H62A	109.00
C3B—C4B—C5B	116.13 (16)	H61A—C6A—H62A	108.00
C4B—C5B—C6B	123.01 (16)	N8A—C9A—H91A	110.00
N5B—C5B—C4B	118.32 (16)	N8A—C9A—H92A	110.00
N5B—C5B—C6B	118.67 (14)	C10A—C9A—H91A	110.00
C1B—C6B—C5B	119.30 (15)	C10A—C9A—H92A	110.00
O11B—C11B—C1B	116.66 (16)	H91A—C9A—H92A	108.00
O11B—C11B—O12B	126.65 (17)	C9A—C10A—H10A	110.00
O12B—C11B—C1B	116.68 (16)	C9A—C10A—H11A	110.00
C3B—C2B—H2B	120.00	C11A—C10A—H10A	110.00
C1B—C2B—H2B	120.00	C11A—C10A—H11A	110.00
C3B—C4B—H4B	122.00	H10A—C10A—H11A	108.00
C5B—C4B—H4B	122.00	N1A—C11A—H12A	109.00
C5B—C6B—H6B	120.00	N1A—C11A—H13A	109.00
C1B—C6B—H6B	120.00	C10A—C11A—H12A	109.00
N1A—C2A—C3A	113.94 (15)	C10A—C11A—H13A	109.00
C2A—C3A—C4A	114.29 (15)	H12A—C11A—H13A	108.00
C3A—C4A—C5A	114.95 (15)	C13A—C12A—H14A	110.00
C4A—C5A—C6A	114.65 (15)	C13A—C12A—H15A	110.00
C5A—C6A—C7A	113.01 (14)	H14A—C12A—H15A	108.00
N1A—C7A—N8A	121.94 (15)	N8A—C13A—H16A	111.00
N1A—C7A—C6A	120.27 (15)	N8A—C13A—H17A	111.00
N8A—C7A—C6A	117.79 (16)	C12A—C13A—H16A	111.00
N8A—C9A—C10A	107.5 (2)	C12A—C13A—H17A	111.00
C9A—C10A—C11A	109.8 (2)	H16A—C13A—H17A	109.00
N1A—C11A—C10A	111.27 (16)	N1A—C11A—H18A	109.00
N8A—C13A—C12A	103.6 (7)	N1A—C11A—H19A	109.00
N1A—C2A—H21A	109.00	C12A—C11A—H18A	109.00
N1A—C2A—H22A	109.00	C12A—C11A—H19A	109.00
C3A—C2A—H21A	109.00	H18A—C11A—H19A	108.00
			
O31B—N3B—C3B—C2B	12.0 (2)	C6B—C1B—C11B—O11B	5.9 (2)
O31B—N3B—C3B—C4B	−168.62 (16)	C6B—C1B—C11B—O12B	−173.03 (16)
O32B—N3B—C3B—C2B	−166.31 (17)	C11B—C1B—C2B—C3B	−175.52 (15)
O32B—N3B—C3B—C4B	13.1 (2)	C2B—C1B—C6B—C5B	−2.6 (2)
O51B—N5B—C5B—C4B	0.3 (2)	C11B—C1B—C6B—C5B	174.94 (15)
O51B—N5B—C5B—C6B	−179.75 (16)	C1B—C2B—C3B—N3B	179.56 (15)
O52B—N5B—C5B—C4B	179.61 (16)	C1B—C2B—C3B—C4B	0.2 (3)
O52B—N5B—C5B—C6B	−0.5 (2)	C2B—C3B—C4B—C5B	−1.7 (2)
C2A—N1A—C11A—C10A	−162.56 (17)	N3B—C3B—C4B—C5B	178.91 (15)
C7A—N1A—C2A—C3A	−71.6 (2)	C3B—C4B—C5B—C6B	1.1 (2)
C11A—N1A—C2A—C3A	110.16 (17)	C3B—C4B—C5B—N5B	−178.96 (15)
C2A—N1A—C7A—N8A	−175.79 (16)	N5B—C5B—C6B—C1B	−178.93 (15)
C2A—N1A—C7A—C6A	5.5 (2)	C4B—C5B—C6B—C1B	1.0 (3)
C11A—N1A—C7A—N8A	2.4 (3)	N1A—C2A—C3A—C4A	78.97 (19)
C11A—N1A—C7A—C6A	−176.35 (15)	C2A—C3A—C4A—C5A	−57.1 (2)
C7A—N1A—C11A—C10A	19.2 (2)	C3A—C4A—C5A—C6A	60.0 (2)
C9A—N8A—C7A—C6A	−173.3 (2)	C4A—C5A—C6A—C7A	−81.00 (19)
C9A—N8A—C7A—N1A	7.9 (3)	C5A—C6A—C7A—N1A	63.5 (2)
C7A—N8A—C9A—C10A	−37.5 (3)	C5A—C6A—C7A—N8A	−115.29 (18)
C6B—C1B—C2B—C3B	2.0 (2)	N8A—C9A—C10A—C11A	55.9 (3)
C2B—C1B—C11B—O11B	−176.60 (16)	C9A—C10A—C11A—N1A	−48.3 (3)
C2B—C1B—C11B—O12B	4.4 (2)		

### (II) Aza-8-azoniabicyclo[5.4.0]undec-7-ene 3,5-dinitrobenzoate Hydrogen-bond geometry (Å, º)

**Table:**

*D*—H···*A*	*D*—H	H···*A*	*D*···*A*	*D*—H···*A*
N8*A*—H8*A*···O11*B*	0.90 (2)	1.88 (2)	2.777 (2)	177 (2)
N8*A*—H8*A*···O12*B*	0.90 (2)	2.53 (2)	3.117 (2)	124 (1)
C10*A*—H11*A*···O32*B*^i^	0.99	2.44	3.247 (3)	138
C11*A*—H13*A*···O52*B*^ii^	0.99	2.52	3.071 (2)	115
C2*A*—H21*A*···O31*B*^iii^	0.99	2.56	3.309 (2)	133
C6*A*—H62*A*···O11*B*	0.99	2.60	3.438 (2)	143
C9*A*—H91*A*···O12*B*	0.99	2.60	3.127 (4)	114

Symmetry codes: (i) −*x*−1/2, *y*+1/2, −*z*+1/2; (ii) *x*+3/2, −*y*+3/2, *z*−1/2; (iii) −*x*+1/2, *y*+1/2, −*z*+1/2.

### (III) 1-Aza-8-azoniabicyclo[5.4.0]undec-7-ene 2-hydroxy-3,5-dinitrobenzoate Crystal data

**Table:**

C_9_H_17_N_2_^+^·C_7_H_3_N_2_O_7_^−^	*F*(000) = 800
*M**_r_* = 380.36	*D*_x_ = 1.489 Mg m^−^^3^
Monoclinic, *P*2_1_/*n*	Mo *K*α radiation, λ = 0.71073 Å
Hall symbol: -P 2yn	Cell parameters from 1891 reflections
*a* = 6.1537 (3) Å	θ = 3.5–26.6°
*b* = 19.1541 (14) Å	µ = 0.12 mm^−^^1^
*c* = 14.5527 (11) Å	*T* = 200 K
β = 98.343 (6)°	Needle, yellow
*V* = 1697.2 (2) Å^3^	0.30 × 0.13 × 0.10 mm
*Z* = 4	

### (III) 1-Aza-8-azoniabicyclo[5.4.0]undec-7-ene 2-hydroxy-3,5-dinitrobenzoate Data collection

**Table:**

Oxford Diffraction Gemini-S CCD-detector diffractometer	3339 independent reflections
Radiation source: Enhance (Mo) X-ray source	2347 reflections with *I* > 2σ(*I*)
Graphite monochromator	*R*_int_ = 0.034
Detector resolution: 16.077 pixels mm^-1^	θ_max_ = 26.0°, θ_min_ = 3.4°
ω scans	*h* = −7→7
Absorption correction: multi-scan (*CrysAlis PRO*; Agilent, 2014)	*k* = −23→23
*T*_min_ = 0.920, *T*_max_ = 0.990	*l* = −17→17
7800 measured reflections	

### (III) 1-Aza-8-azoniabicyclo[5.4.0]undec-7-ene 2-hydroxy-3,5-dinitrobenzoate Refinement

**Table:**

Refinement on *F*^2^	Primary atom site location: structure-invariant direct methods
Least-squares matrix: full	Secondary atom site location: difference Fourier map
*R*[*F*^2^ > 2σ(*F*^2^)] = 0.058	Hydrogen site location: inferred from neighbouring sites
*wR*(*F*^2^) = 0.123	H atoms treated by a mixture of independent and constrained refinement
*S* = 1.03	*w* = 1/[σ^2^(*F*_o_^2^) + (0.0374*P*)^2^ + 0.7569*P*] where *P* = (*F*_o_^2^ + 2*F*_c_^2^)/3
3339 reflections	(Δ/σ)_max_ < 0.001
263 parameters	Δρ_max_ = 0.29 e Å^−^^3^
3 restraints	Δρ_min_ = −0.29 e Å^−^^3^

### (III) 1-Aza-8-azoniabicyclo[5.4.0]undec-7-ene 2-hydroxy-3,5-dinitrobenzoate Special details

**Table:**

Geometry. Bond distances, angles etc. have been calculated using the rounded fractional coordinates. All su's are estimated from the variances of the (full) variance-covariance matrix. The cell esds are taken into account in the estimation of distances, angles and torsion angles
Refinement. Refinement of F^2^ against ALL reflections. The weighted R-factor wR and goodness of fit S are based on F^2^, conventional R-factors R are based on F, with F set to zero for negative F^2^. The threshold expression of F^2^ > 2sigma(F^2^) is used only for calculating R-factors(gt) etc. and is not relevant to the choice of reflections for refinement. R-factors based on F^2^ are statistically about twice as large as those based on F, and R- factors based on ALL data will be even larger.

### (III) 1-Aza-8-azoniabicyclo[5.4.0]undec-7-ene 2-hydroxy-3,5-dinitrobenzoate Fractional atomic coordinates and isotropic or equivalent isotropic displacement parameters (Å^2^)

**Table:**

	*x*	*y*	*z*	*U*_iso_*/*U*_eq_	Occ. (<1)
O2B	0.8426 (4)	0.56153 (13)	0.78929 (15)	0.0433 (8)	0.720
O11B	0.5084 (3)	0.68879 (9)	0.59293 (13)	0.0433 (6)	
O12B	0.5450 (3)	0.64525 (10)	0.73596 (13)	0.0522 (7)	
O31B	1.1116 (4)	0.45700 (12)	0.81707 (17)	0.0819 (10)	
O32B	1.4080 (4)	0.47261 (13)	0.75765 (15)	0.0761 (9)	
O51B	1.3286 (3)	0.55867 (11)	0.44585 (14)	0.0594 (7)	
O52B	1.0707 (4)	0.63206 (11)	0.39870 (14)	0.0670 (8)	
N3B	1.2118 (4)	0.48306 (12)	0.76028 (16)	0.0467 (8)	
N5B	1.1654 (3)	0.59169 (11)	0.45698 (15)	0.0407 (7)	
C1B	0.8002 (3)	0.60950 (11)	0.63899 (16)	0.0268 (7)	
C2B	0.9062 (3)	0.56600 (12)	0.70947 (16)	0.0297 (7)	
C3B	1.0956 (4)	0.53052 (12)	0.69146 (16)	0.0310 (7)	
C4B	1.1816 (3)	0.53943 (11)	0.61041 (16)	0.0308 (7)	
C5B	1.0735 (3)	0.58226 (11)	0.54278 (15)	0.0276 (7)	
C6B	0.8810 (3)	0.61671 (11)	0.55531 (15)	0.0263 (7)	
C11B	0.6029 (4)	0.65080 (12)	0.65595 (19)	0.0346 (8)	
O21B	0.7762 (10)	0.6571 (3)	0.4915 (5)	0.052 (3)	0.280
N1A	−0.1524 (3)	0.82026 (10)	0.63820 (13)	0.0293 (6)	
N8A	0.1714 (3)	0.76040 (11)	0.67301 (14)	0.0369 (7)	
C2A	−0.3262 (3)	0.85087 (13)	0.56984 (17)	0.0357 (8)	
C3A	−0.2606 (4)	0.91805 (13)	0.52684 (18)	0.0397 (8)	
C4A	−0.1188 (4)	0.90797 (14)	0.45044 (17)	0.0409 (8)	
C5A	0.0934 (4)	0.86814 (13)	0.48033 (17)	0.0393 (9)	
C6A	0.0612 (4)	0.79368 (13)	0.51409 (16)	0.0340 (8)	
C7A	0.0226 (3)	0.79083 (11)	0.61294 (15)	0.0265 (7)	
C9A	0.1399 (9)	0.7478 (2)	0.7696 (4)	0.0366 (18)	0.686 (4)
C10A	0.0234 (6)	0.8111 (2)	0.8005 (3)	0.0379 (11)	0.686 (4)
C11A	−0.1871 (4)	0.82349 (13)	0.73612 (16)	0.0363 (8)	
C13A	0.189 (2)	0.7738 (7)	0.7752 (11)	0.0366 (18)	0.314 (4)
C12A	−0.0464 (13)	0.7704 (5)	0.7958 (6)	0.0379 (11)	0.314 (4)
H4B	1.31350	0.51650	0.60110	0.0370*	
H6B	0.80240	0.64380	0.50700	0.0320*	0.720
H2B	0.73870	0.58950	0.79190	0.0650*	0.720
H21B	0.66080	0.67200	0.50930	0.0770*	0.280
H61B	0.85460	0.56120	0.76770	0.0360*	0.280
H8A	0.280 (3)	0.7394 (11)	0.6508 (15)	0.0320*	
H10A	−0.00890	0.80380	0.86450	0.0460*	0.686 (4)
H21A	−0.45670	0.85990	0.60060	0.0430*	
H22A	−0.36910	0.81640	0.51980	0.0430*	
H31A	−0.17940	0.94750	0.57630	0.0480*	
H32A	−0.39530	0.94360	0.50080	0.0480*	
H41A	−0.20570	0.88270	0.39820	0.0490*	
H42A	−0.08210	0.95440	0.42720	0.0490*	
H51A	0.18300	0.89440	0.53080	0.0470*	
H52A	0.17720	0.86610	0.42730	0.0470*	
H61A	−0.06570	0.77230	0.47440	0.0410*	
H62A	0.19310	0.76570	0.50720	0.0410*	
H91A	0.28350	0.74140	0.80920	0.0440*	0.686 (4)
H92A	0.05040	0.70540	0.77390	0.0440*	0.686 (4)
H11A	0.11950	0.85260	0.80070	0.0460*	0.686 (4)
H12A	−0.29640	0.78780	0.74760	0.0440*	0.686 (4)
H13A	−0.24650	0.86990	0.74910	0.0440*	0.686 (4)
H14A	−0.10610	0.72290	0.78230	0.0460*	0.314 (4)
H15A	−0.04950	0.78040	0.86230	0.0460*	0.314 (4)
H16A	0.25340	0.82040	0.79120	0.0440*	0.314 (4)
H17A	0.28080	0.73790	0.81100	0.0440*	0.314 (4)
H18A	−0.34390	0.81460	0.74010	0.0440*	0.314 (4)
H19A	−0.15090	0.87100	0.76060	0.0440*	0.314 (4)

### (III) 1-Aza-8-azoniabicyclo[5.4.0]undec-7-ene 2-hydroxy-3,5-dinitrobenzoate Atomic displacement parameters (Å^2^)

**Table:**

	*U*^11^	*U*^22^	*U*^33^	*U*^12^	*U*^13^	*U*^23^
O2B	0.0472 (13)	0.0563 (16)	0.0297 (14)	0.0095 (12)	0.0168 (11)	0.0083 (12)
O11B	0.0356 (9)	0.0369 (10)	0.0579 (12)	0.0124 (8)	0.0087 (8)	0.0063 (9)
O12B	0.0492 (11)	0.0640 (13)	0.0484 (12)	0.0080 (10)	0.0245 (9)	−0.0040 (10)
O31B	0.0774 (15)	0.0847 (18)	0.0749 (17)	−0.0226 (13)	−0.0181 (12)	0.0525 (14)
O32B	0.0708 (15)	0.0894 (18)	0.0629 (15)	0.0492 (13)	−0.0082 (11)	0.0045 (12)
O51B	0.0542 (11)	0.0636 (13)	0.0680 (14)	0.0066 (10)	0.0346 (10)	−0.0115 (11)
O52B	0.0918 (15)	0.0703 (15)	0.0456 (13)	0.0168 (13)	0.0323 (11)	0.0224 (11)
N3B	0.0592 (15)	0.0338 (13)	0.0411 (14)	0.0003 (11)	−0.0127 (12)	0.0001 (11)
N5B	0.0464 (12)	0.0388 (13)	0.0405 (13)	−0.0040 (10)	0.0189 (10)	−0.0056 (11)
C1B	0.0259 (11)	0.0216 (12)	0.0323 (13)	−0.0038 (9)	0.0021 (9)	−0.0035 (10)
C2B	0.0341 (12)	0.0270 (13)	0.0280 (13)	−0.0057 (10)	0.0044 (10)	−0.0019 (10)
C3B	0.0361 (12)	0.0241 (12)	0.0300 (14)	−0.0006 (10)	−0.0049 (10)	0.0015 (10)
C4B	0.0256 (11)	0.0245 (12)	0.0406 (15)	−0.0010 (10)	−0.0006 (10)	−0.0054 (11)
C5B	0.0297 (11)	0.0257 (12)	0.0285 (13)	−0.0056 (10)	0.0077 (10)	−0.0023 (10)
C6B	0.0288 (11)	0.0214 (12)	0.0275 (13)	−0.0017 (9)	−0.0001 (9)	0.0021 (10)
C11B	0.0292 (12)	0.0284 (13)	0.0466 (16)	−0.0028 (10)	0.0068 (11)	−0.0055 (12)
O21B	0.043 (4)	0.059 (5)	0.053 (4)	0.008 (3)	0.009 (3)	0.025 (4)
N1A	0.0254 (9)	0.0319 (11)	0.0304 (11)	0.0014 (8)	0.0038 (8)	−0.0007 (9)
N8A	0.0333 (11)	0.0476 (13)	0.0299 (12)	0.0157 (10)	0.0051 (9)	0.0034 (10)
C2A	0.0254 (11)	0.0378 (14)	0.0427 (15)	0.0059 (10)	0.0006 (10)	−0.0026 (12)
C3A	0.0358 (13)	0.0339 (14)	0.0464 (16)	0.0066 (11)	−0.0044 (11)	0.0003 (12)
C4A	0.0442 (14)	0.0384 (15)	0.0365 (15)	−0.0024 (12)	−0.0061 (11)	0.0074 (12)
C5A	0.0370 (13)	0.0508 (17)	0.0308 (14)	−0.0005 (12)	0.0075 (10)	0.0080 (12)
C6A	0.0340 (12)	0.0413 (15)	0.0262 (13)	0.0081 (11)	0.0029 (10)	−0.0052 (11)
C7A	0.0270 (11)	0.0226 (12)	0.0291 (13)	−0.0006 (9)	0.0014 (9)	−0.0035 (10)
C9A	0.042 (3)	0.037 (4)	0.0292 (18)	0.001 (2)	0.000 (2)	0.005 (3)
C10A	0.047 (2)	0.041 (2)	0.0263 (17)	−0.0052 (17)	0.0070 (16)	−0.0033 (19)
C11A	0.0363 (13)	0.0419 (15)	0.0335 (14)	−0.0014 (11)	0.0150 (11)	−0.0058 (12)
C13A	0.042 (3)	0.037 (4)	0.0292 (18)	0.001 (2)	0.000 (2)	0.005 (3)
C12A	0.047 (2)	0.041 (2)	0.0263 (17)	−0.0052 (17)	0.0070 (16)	−0.0033 (19)

### (III) 1-Aza-8-azoniabicyclo[5.4.0]undec-7-ene 2-hydroxy-3,5-dinitrobenzoate Geometric parameters (Å, º)

**Table:**

O2B—C2B	1.281 (3)	C3A—C4A	1.522 (4)
O11B—C11B	1.247 (3)	C4A—C5A	1.520 (4)
O12B—C11B	1.271 (3)	C5A—C6A	1.531 (4)
O21B—C6B	1.305 (7)	C6A—C7A	1.493 (3)
O31B—N3B	1.208 (3)	C9A—C10A	1.510 (6)
O32B—N3B	1.230 (4)	C10A—C11A	1.503 (5)
O51B—N5B	1.217 (3)	C12A—C13A	1.523 (15)
O52B—N5B	1.230 (3)	C2A—H21A	0.9900
O2B—H2B	0.8400	C2A—H22A	0.9900
O21B—H21B	0.8400	C3A—H31A	0.9900
N3B—C3B	1.460 (3)	C3A—H32A	0.9900
N5B—C5B	1.455 (3)	C4A—H41A	0.9900
N1A—C2A	1.473 (3)	C4A—H42A	0.9900
N1A—C11A	1.473 (3)	C5A—H51A	0.9900
N1A—C7A	1.314 (3)	C5A—H52A	0.9900
N8A—C9A	1.467 (6)	C6A—H61A	0.9900
N8A—C13A	1.498 (16)	C6A—H62A	0.9900
N8A—C7A	1.308 (3)	C9A—H91A	0.9900
N8A—H8A	0.88 (2)	C9A—H92A	0.9900
C1B—C11B	1.499 (3)	C10A—H10A	0.9900
C1B—C6B	1.387 (3)	C10A—H11A	0.9900
C1B—C2B	1.406 (3)	C11A—H12A	0.9900
C2B—C3B	1.406 (3)	C11A—H13A	0.9900
C3B—C4B	1.372 (3)	C12A—H14A	0.9900
C4B—C5B	1.376 (3)	C12A—H15A	0.9900
C5B—C6B	1.391 (3)	C13A—H16A	0.9900
C2B—H61B	0.9500	C13A—H17A	0.9900
C4B—H4B	0.9500	C11A—H18A	0.9900
C6B—H6B	0.9500	C11A—H19A	0.9900
C2A—C3A	1.511 (3)		
			
C2B—O2B—H2B	109.00	C9A—C10A—C11A	110.2 (3)
C6B—O21B—H21B	110.00	N1A—C11A—C10A	111.3 (2)
O32B—N3B—C3B	117.7 (2)	N8A—C13A—C12A	104.7 (9)
O31B—N3B—O32B	123.7 (2)	N1A—C2A—H21A	109.00
O31B—N3B—C3B	118.6 (2)	N1A—C2A—H22A	109.00
O52B—N5B—C5B	117.7 (2)	C3A—C2A—H21A	109.00
O51B—N5B—C5B	118.7 (2)	C3A—C2A—H22A	109.00
O51B—N5B—O52B	123.5 (2)	H21A—C2A—H22A	108.00
C2A—N1A—C11A	116.36 (18)	C2A—C3A—H31A	109.00
C7A—N1A—C11A	121.90 (19)	C2A—C3A—H32A	109.00
C2A—N1A—C7A	121.74 (19)	C4A—C3A—H31A	109.00
C7A—N8A—C13A	122.0 (5)	C4A—C3A—H32A	109.00
C7A—N8A—C9A	122.5 (3)	H31A—C3A—H32A	108.00
C9A—N8A—H8A	119.3 (14)	C3A—C4A—H41A	109.00
C13A—N8A—H8A	119.6 (15)	C3A—C4A—H42A	109.00
C7A—N8A—H8A	117.0 (14)	C5A—C4A—H41A	109.00
C2B—C1B—C6B	120.78 (18)	C5A—C4A—H42A	109.00
C2B—C1B—C11B	119.6 (2)	H41A—C4A—H42A	108.00
C6B—C1B—C11B	119.6 (2)	C4A—C5A—H51A	109.00
O2B—C2B—C1B	122.0 (2)	C4A—C5A—H52A	109.00
C1B—C2B—C3B	117.4 (2)	C6A—C5A—H51A	109.00
O2B—C2B—C3B	120.5 (2)	C6A—C5A—H52A	109.00
N3B—C3B—C4B	117.1 (2)	H51A—C5A—H52A	108.00
C2B—C3B—C4B	122.3 (2)	C5A—C6A—H61A	109.00
N3B—C3B—C2B	120.6 (2)	C5A—C6A—H62A	109.00
C3B—C4B—C5B	118.84 (19)	C7A—C6A—H61A	109.00
C4B—C5B—C6B	121.43 (19)	C7A—C6A—H62A	109.00
N5B—C5B—C4B	118.69 (18)	H61A—C6A—H62A	108.00
N5B—C5B—C6B	119.88 (19)	N8A—C9A—H91A	110.00
O21B—C6B—C1B	118.7 (3)	N8A—C9A—H92A	110.00
O21B—C6B—C5B	122.0 (3)	C10A—C9A—H91A	110.00
C1B—C6B—C5B	119.23 (19)	C10A—C9A—H92A	110.00
O12B—C11B—C1B	116.6 (2)	H91A—C9A—H92A	109.00
O11B—C11B—C1B	119.3 (2)	C9A—C10A—H10A	110.00
O11B—C11B—O12B	124.1 (2)	C9A—C10A—H11A	110.00
C3B—C2B—H61B	121.00	C11A—C10A—H10A	110.00
C1B—C2B—H61B	122.00	C11A—C10A—H11A	110.00
C5B—C4B—H4B	121.00	H10A—C10A—H11A	108.00
C3B—C4B—H4B	121.00	N1A—C11A—H12A	109.00
C1B—C6B—H6B	120.00	N1A—C11A—H13A	109.00
C5B—C6B—H6B	121.00	C10A—C11A—H12A	109.00
N1A—C2A—C3A	114.04 (18)	C10A—C11A—H13A	109.00
C2A—C3A—C4A	114.2 (2)	H12A—C11A—H13A	108.00
C3A—C4A—C5A	114.5 (2)	C13A—C12A—H15A	110.00
C4A—C5A—C6A	114.4 (2)	H14A—C12A—H15A	108.00
C5A—C6A—C7A	112.9 (2)	N8A—C13A—H16A	111.00
N1A—C7A—N8A	121.8 (2)	N8A—C13A—H17A	111.00
N1A—C7A—C6A	120.35 (19)	C12A—C13A—H16A	111.00
N8A—C7A—C6A	117.82 (19)	C12A—C13A—H17A	111.00
N8A—C9A—C10A	106.7 (3)	H16A—C13A—H17A	109.00
			
O31B—N3B—C3B—C2B	23.9 (3)	C6B—C1B—C11B—O11B	3.1 (3)
O31B—N3B—C3B—C4B	−157.2 (2)	C6B—C1B—C11B—O12B	−175.8 (2)
O32B—N3B—C3B—C2B	−155.2 (2)	C2B—C1B—C11B—O11B	−179.4 (2)
O32B—N3B—C3B—C4B	23.8 (3)	C2B—C1B—C11B—O12B	1.8 (3)
O51B—N5B—C5B—C4B	3.7 (3)	C11B—C1B—C6B—C5B	175.2 (2)
O51B—N5B—C5B—C6B	−176.8 (2)	O2B—C2B—C3B—N3B	5.6 (4)
O52B—N5B—C5B—C4B	−177.5 (2)	O2B—C2B—C3B—C4B	−173.3 (2)
O52B—N5B—C5B—C6B	2.0 (3)	C1B—C2B—C3B—N3B	−178.5 (2)
C2A—N1A—C7A—N8A	−176.4 (2)	C1B—C2B—C3B—C4B	2.7 (3)
C2A—N1A—C7A—C6A	6.0 (3)	C2B—C3B—C4B—C5B	−2.9 (3)
C11A—N1A—C7A—N8A	2.7 (3)	N3B—C3B—C4B—C5B	178.3 (2)
C11A—N1A—C7A—C6A	−175.0 (2)	C3B—C4B—C5B—C6B	0.4 (3)
C2A—N1A—C11A—C10A	−163.0 (2)	C3B—C4B—C5B—N5B	179.9 (2)
C7A—N1A—C2A—C3A	−71.7 (3)	N5B—C5B—C6B—C1B	−177.36 (19)
C11A—N1A—C2A—C3A	109.2 (2)	C4B—C5B—C6B—C1B	2.1 (3)
C7A—N1A—C11A—C10A	17.9 (3)	N1A—C2A—C3A—C4A	78.8 (3)
C7A—N8A—C9A—C10A	−38.8 (4)	C2A—C3A—C4A—C5A	−57.5 (3)
C9A—N8A—C7A—C6A	−173.2 (3)	C3A—C4A—C5A—C6A	61.0 (3)
C9A—N8A—C7A—N1A	9.1 (4)	C4A—C5A—C6A—C7A	−82.0 (3)
C6B—C1B—C2B—C3B	0.0 (3)	C5A—C6A—C7A—N1A	63.3 (3)
C11B—C1B—C2B—O2B	−1.6 (3)	C5A—C6A—C7A—N8A	−114.5 (2)
C11B—C1B—C2B—C3B	−177.5 (2)	N8A—C9A—C10A—C11A	56.2 (4)
C2B—C1B—C6B—C5B	−2.3 (3)	C9A—C10A—C11A—N1A	−47.7 (4)
C6B—C1B—C2B—O2B	175.9 (2)		

### (III) 1-Aza-8-azoniabicyclo[5.4.0]undec-7-ene 2-hydroxy-3,5-dinitrobenzoate Hydrogen-bond geometry (Å, º)

**Table:**

*D*—H···*A*	*D*—H	H···*A*	*D*···*A*	*D*—H···*A*
N8*A*—H8*A*···O11*B*	0.88 (2)	1.99 (2)	2.871 (3)	176 (2)
O2*B*—H2*B*···O12*B*	0.84	1.72	2.473 (3)	149
C10*A*—H11*A*···O32*B*^i^	0.99	2.45	3.251 (5)	138
C11*A*—H13*A*···O52*B*^ii^	0.99	2.59	3.093 (3)	111
C2*A*—H21*A*···O31*B*^iii^	0.99	2.48	3.281 (3)	138

Symmetry codes: (i) −*x*+3/2, *y*+1/2, −*z*+3/2; (ii) *x*−3/2, −*y*+3/2, *z*+1/2; (iii) −*x*+1/2, *y*+1/2, −*z*+3/2.
